# E-Cigarette Use among Male Smokers in Al-Ahsa, Kingdom of Saudi Arabia: A Cross-Sectional Study

**DOI:** 10.3390/ijerph20010143

**Published:** 2022-12-22

**Authors:** Ahmed M. Al Rajeh, Ilias Mahmud, Mahmudul Hassan Al Imam, Muhammad Aziz Rahman, Fariss Al Shehri, Salman Alomayrin, Nawaf Alfazae, Yousif Mohammed Elmosaad, Ibrahim Alasqah

**Affiliations:** 1 Department of Respiratory Care, College of Applied Medical Sciences, King Faisal University, Al-Ahsa 31982, Saudi Arabia; 2 Department of Public Health, College of Public Health and Health Informatics, Qassim University, Al-Bukairiyah 52741, Saudi Arabia; 3 School of Health, Medical, and Applied Sciences, Central Queensland University, Rockhampton, QLD 4701, Australia; 4 Central Queensland Public Health Unit, Central Queensland Hospital and Health Service, Rockhampton, QLD 4701, Australia; 5 Institute of Health and Wellbeing, Federation University Australia, Berwick, VIC 3350, Australia; 6 Australian Institute for Primary Care & Ageing (AIPCA), La Trobe University, Melbourne, VIC 3086, Australia; 7 Department of Public Health, College of Applied Medical Sciences, King Faisal University, Al-Ahsa 31982, Saudi Arabia

**Keywords:** e-cigarette, cigarette, smoking, Saudi Arabia

## Abstract

E-cigarette use is increasing globally. Recent evidence suggests that e-cigarettes contain harmful substances that could cause adverse health outcomes. This study investigated the prevalence and associated factors of e-cigarette use among male current smokers in Saudi Arabia. We conducted a cross-sectional survey of adult male current smokers in the Al-Ahsa province of Saudi Arabia. Data were collected using a structured questionnaire. We performed logistic regression analyses to investigate the factors associated with e-cigarette use among adult male current smokers. 325 current smokers participated in the study. A third of them (33.5%) were e-cigarette users. Almost all the study participants (97.0%) had heard about e-cigarettes. Participants who were occasional smokers (Odds Ratio (OR): 2.28; 95% Confidence Interval (CI): 1.17–4.41) and had good knowledge perception of e-cigarettes (OR 3.49; 95% CI: 2.07–5.90) had higher odds of using e-cigarettes when compared to regular smokers of conventional cigarettes and current smokers with poor knowledge perception of e-cigarettes, respectively. In contrast, private employees (OR: 0.25, 95% CI: 0.07–0.85), and business owners (OR: 0.09, 95% CI: 0.01–0.63) had lower odds of using e-cigarettes compared to unemployed individuals. Compared with non-e-cigarette users, the rate of conventional cigarette smoking per day was significantly lower among e-cigarette users. Use of e-cigarette (OR: 3.57, 95% CI: 2.14–5.98), believing that e-cigarette quitting is hard (OR: 2.02, 95% CI: 1.17–3.49) and trying to quit e-cigarettes (OR: 2.17, 95% CI: 1.1–4.25) were found to be significant predictors of good knowledge perception of e-cigarettes among the current smokers. The use and knowledge perception of e-cigarettes were higher among occasional conventional male cigarette smokers than regular male smokers in Al-Ahsa province. The use of e-cigarettes as smoking cessation aids should be examined further in the Saudi Arabian setting.

## 1. Introduction

Globally, electronic cigarette (e-cigarette) use has increased rapidly, particularly among teenagers and young adults who have never smoked [1]. Over the past few years, e-cigarette use has become common among current smokers [2]. E-cigarettes are delivered by an electronic device, which heats and aerosolizes a liquid solution of nicotine, propylene glycol, vegetable glycerin, and flavoring additives [3]. E-cigarette manufacturers promote it as a safer and cheaper alternative to conventional cigarette smoking [4], and the media play a major role in convincing people to believe this argument [5]. Previous studies in the Kingdom of Saudi Arabia (KSA) have estimated the prevalence of e-cigarette use at 27.7% among university students in the city of Jeddah [6] and 10.6% in the Qassim region [7], regardless of their conventional cigarette smoking status, while 12.2% in Riyadh among smoker students [8]. Data from the United States of America (USA), Canada, Europe, and New Zealand showed that e-cigarette use has increased rapidly over the past few years [9,10]. Prior evidence suggests that e-cigarettes are primarily used by middle-aged current smokers, particularly males, to quit smoking or for recreation [3]. 

The scientific community is divided about the use of e-cigarettes as a smoking cessation tool [11]. A 6-month prospective study from Italy showed that e-cigarette use is associated with an increased likelihood of smoking cessation as, 32.5% of the participants reduced their cigarette consumption by at least 50% [12]. Another study reported that e-cigarette users have more desire to quit smoking in France [13]. A recent randomized controlled trial showed the effectiveness of e-cigarettes over conventional nicotine-replacement therapies for smoking cessation in the United Kingdom [14]. Moreover, a Cochrane review and a systematic review concluded that nicotine e-cigarettes help smokers stop smoking in the long term [3,15]. A Cochrane review of randomized controlled trials found moderate evidence of the impact of e-cigarette use in helping smokers with long-term quitting [16]. On the other hand, a meta-analysis reported that e-cigarette users had a 28% lower likelihood of quitting smoking [17], and Kim et al. [15] reported that e-cigarette use does not have a causal effect on concurrent conventional smoking among young adults in the USA. Another recent systematic review and meta-analysis by Hedman et al. [18] reported a lack of evidence for an association between e-cigarette use and smoking cessation. Another meta-analysis also concluded that e-cigarettes are not associated with increased smoking cessation in the adult population [19]. Therefore, the efficacy of e-cigarette use as a smoking cessation tool is not conclusive.

In this context, the high prevalence of e-cigarette use and associated safety data have raised public-health concerns [20,21,22]. Although some studies have shown that using e-cigarettes is considerably less harmful than smoking combustible cigarettes [23,24], recent evidence suggests that e-cigarettes contain harmful substances that could cause adverse health outcomes. Jankowski et al. [25] reported that e-cigarettes include a range of potentially hazardous substances, such as formaldehyde, acetaldehyde, acrolein, propanol, nicotine, acetone, o-methyl benzaldehyde, carcinogenic nitrosamines, and carcinogenic metals, which are toxic to cells. In addition to that, the flavorings in e-cigarettes containing lung toxins, such as diacetyl, diketones, and menthol, contributed more to cell mortality than tobacco flavoring [26,27]. A European study stated that there is no evidence that e-cigarettes could be safer than conventional cigarettes in the long term [28]. Moreover, other studies supported that e-cigarettes could not be considered harmless, despite being less noxious than conventional tobacco cigarettes [24,29]. 

In the KSA, the retail sale of e-cigarettes is allowed. There are no restrictions on advertising, promotion, and sponsorship of e-cigarettes (Supreme Decree No. 38621 dated 11/7/1440 AH (18 March 2019)). E-cigarettes have become popular among adults in the country [6]. However, there is limited evidence on the use of e-cigarettes, their relation to conventional tobacco smoking, and associated socio-demographic factors of e-cigarette smoking in the KSA. Considering the importance of tobacco control and potential harm-reduction strategies, it is important to examine these issues in the KSA. Therefore, we aimed to assess the use of e-cigarettes among male current smokers, examine the association between e-cigarette use and cigarette smoking patterns, and identify factors associated with the use of e-cigarettes among cigarette smokers in the KSA.

## 2. Methods

### 2.1. Study Design and Settings

We conducted a cross-sectional survey of male customers of cafes in Al-Ahsa, KSA, between September 2019 and December 2019. The Al-Ahsa province includes three administrative areas, Al-Hofuf, Al-Mubaraz, and Al-Gourah. According to the Central Authority for Statistics, the population of this province is 1,041,863 of which 525,100 are aged 18 years or more. Among the adults, 42.0%, 38.0%, and 20% were living in Al Hofuf, Al-Mubaraz, and Al-Gourah, respectively.

### 2.2. Study Population and Sampling

All adult male current smokers residing in Al-Ahsa and customers at cafes were considered as the study population. The majority of the customers at these cafes are young and middle-aged individuals. In Saudi culture, cafes are popular places for smoking as well as for socialization. Although in the KSA, smoking is prohibited inside a cafe, the usual practice is to smoke outside. The inclusion criteria of this study were adult males (≥18 years), who reported smoking conventional cigarettes during the last 30 days. Adult males currently smoking but who did not wish to participate in the study, smokers from outside the Al-Ahsa, and adult males who use e-cigarettes, but are not current smokers were excluded.

As the estimated prevalence of smoking among adults in the KSA is 12.01% [30], the total number of smokers in Al-Ahsa province was estimated to be 63,064. A sample size of 325 adult current smokers was obtained using the Leslie Kish formula: n = Z^2^PQ/D2, where Z is the corresponding value to 95% confidence limits = 1.96, P = prevalence rate of smoking (0.12), Q = I − P = (0.88), D = desired margin of error (absolute precision) = 0.05, and effect size = 2. The total sample size (325) was distributed proportionally for each administrative area—136 (42.0%) participants from Al-Hofuf, 124 (38.0%) from Al-Mubaraz, and 65 (20.0%) from Al-Gourah. 

A multistage probability sampling technique was used to select a representative sample from the study population. First, a list of 151 coffee shops (46 coffee shops in Al-Hofuf, 41 in Al-Mubarraz, and 16 in Al-Gourah) was obtained from the Al-Ahsa municipality. Out of these 151 coffee shops, 50% were randomly selected and distributed proportionately—23 from Al-Hofuf, 21 from Al-Mubaraz, and 8 from Al-Gourah.

To determine the required number of participants from each coffee shop, the number of selected coffee shops was divided by the sample size of the administrative area. For example, from Al-Hofuf, six participants (136/23) were selected randomly from each coffee shop. Participants’ details are presented later (Table 1, Table 2, Table 3 and Table 4). 

### 2.3. Data Collection

Data were collected through a 10–15-min face-to-face interview using a structured questionnaire, which had three sections. Section 1 included questions related to the socio-demographic characteristics of the participants, such as age, educational level, occupation, marital status, and monthly income. Section 2 included questions related to conventional cigarette smoking status, smoking pattern, number of cigarettes per day, the duration of the smoking habit, and the reasons for smoking. Section 3 included questions assessing knowledge of e-cigarettes, e-cigarette usage habits, the duration and frequency of use, and motivation behind e-cigarette usage.

### 2.4. Definition of Study Variables

#### 2.4.1. Smoking 

Smokers were asked about their smoking behavior to confirm their current smoking status with the following survey items: “Have you smoked cigarettes during the past 30 days?”. If they reported, “Yes”, they were classified as “smokers”, and “Do you now smoke cigarettes regularly, or occasionally?”, and the duration and frequency of their use. We distinguished those declaring being regular smokers and had smoked more than 24 cigarettes per day. These were classified as “heavy smokers”. The participants were asked to select the main reasons that encouraged them to be smokers from a list of several options. Smoking cessation attempts were assessed by asking them, “Have you tried to quit smoking in the past 30 days?” (Yes/No).

#### 2.4.2. The Use of E-Cigarettes

The use of e-cigarettes was assessed by asking smokers “Have you used e-cigarettes in the past 30 days?” (Yes/No). If a participant answered “Yes”, he was marked as a current user. In addition, participants were asked, “Which of the following statements best describes you?” The response options included “regular users" and “occasional users”. To assess the intensity and frequency of their use of e-cigarettes, they were asked this question: “How many hours per day do you use e-cigarettes?”. Moreover, the participants who indicated using e-cigarettes were asked, "Do you feel e-cigarettes help you to reduce the number of cigarettes you smoke?” (Yes/No). If a participant answered “Yes”, then they were asked: “How many cigarettes have you reduced while using e-cigarettes?”. Participants who indicated using e-cigarettes were asked: “What are the reasons for using e-cigarettes?”. In addition, the participants were asked to identify whether they intended to use e-cigarettes as a tool to quit smoking. Participants currently using both electronic and conventional cigarettes were classified as “dual users”. To determine the e-cigarette addiction characteristics, the users were asked to identify whether they tried to stop the use of e-cigarettes during the past 30 days. The response options were provided as “Yes/No”. If a participant answered “No”, he was then asked: “Is the e-cigarette use harder to quit?” The response options were provided in “Yes/No”.

#### 2.4.3. Knowledge of E-Cigarettes

Participants’ perceived knowledge was assessed through a set of seven questions, which were adopted from previous studies [31,32,33]. The participants were asked the following: “Have you heard of the electronic cigarette?” (Yes/No); “Do you know how electronic cigarettes work?” (Yes/No); “Do you know different e-cigarette brands?” (Yes/No); “Do you know the various ingredients in e-cigarettes?” (Yes/No); “Are you aware of the nicotine levels in e-cigarettes?” (Yes/No); “Do you know the health consequences of e-cigarette use in the long term?” (Yes/No); “Does the electronic cigarette have addictive properties?” (Yes/No). A composite variable was generated to summarize the overall participant’s perceived knowledge about e-cigarettes and was calculated by adding up the score for each of all the questions, and the maximum possible score was 7 points. The question answered with “Yes” was given one point and zero points if the answer was “No”.

The scores of the knowledge were dichotomized by assuming a cutoff of 75% [34]. Based on that, a participant who answered “yes” to 75% or more of the knowledge questions was considered to have a good perception level of knowledge of e-cigarettes.

### 2.5. Data Analysis

The collected data were entered and analyzed using SPSS (Statistical Package for the Social Sciences) version 20. Data were entered by two different researchers to ensure accuracy. For continuous variables, the mean (±standard deviation) was estimated. Categorical variables were analyzed using proportions, which included an assessment of the perceived knowledge of e-cigarettes. Chi-square tests were used to determine the association between the use of e-cigarettes and smoking patterns. Logistic regression analyses were used to identify factors associated with e-cigarette use; odds ratios (ORs) with 95% confidence intervals (CIs) were reported. Multivariate logistic regression was used to control the potential confounders, such as age and occupation; adjusted ORs (aORs) and 95% CIs were reported. Statistical significance was indicated by *p* < 0.05.

## 3. Results

A total of 325 smokers participated in the study and the mean age was 24.7 (±5.1) years. The majority of them (76.9%; n = 250) were young (18–27 years old). 

### 3.1. Smoking Status

Table 1 illustrates that nearly three-quarters (73.5%; n = 239) of respondents who described themselves as regular smokers, and 69.8% (n = 227) were smoking 9–24 cigarettes per day. The average number of cigarettes smoked per day was 16.8 ± 8.9. The primary, self-reported, reason for smoking initiation was peer effect (55.7%; n = 181), followed by stress relief (38.8%; n = 126) and enjoyment (36.6%; n = 119). About half of the regular or occasional smokers (49.1%; n = 160) attempted to quit smoking within the past 30 days, and over half of them (50.5%; n = 164) reported smoking for the past 6 years or more.

**Table 1:** Conventional cigarette-smoking status of the participants (n = 325).

Variables	n	%
Current smokers		
Occasional smokers	86	26.5
Regular smokers	239	73.5
Number of cigarettes smoked per day		
<9 cigarettes	50	15.4
9–16 cigarettes	112	34.5
17–24 cigarettes	115	35.4
>24 cigarettes	48	14.8
Years of smoking		
<6 years	161	49.5
6–10 years	92	28.3
11–15 years	53	16.3
>15 years	19	8.5
Reasons for smoking initiation (not mutually exclusive)		
Smokers in the family	72	22.2
Peer effects	181	55.7
Sadness	75	23.1
Stress	126	38.8
Entertainment	119	36.6
Tried to quit smoking		
No	165	50.8
Yes	160	49.2

### 3.2. Perceived Knowledge of E-Cigarette

Table 2 shows that the majority of the participants reported that they were aware of e-cigarettes (n = 315; 96.9%) and their different brands (n = 282; 86.8%); knew how e-cigarettes work (n = 223; 68.6%); knew the health consequences of e-cigarette use (n = 196; 60.3%); ingredients in e-cigarettes (n = 175; 53.8%); and the nicotine level in e-cigarettes (n = 221; 68.0%). Only 36.3% (n = 118) reported that they were aware of the addictive properties of e-cigarettes. All variables but the awareness of the health consequences of e-cigarette use were significantly associated with perceived knowledge of e-cigarettes and e-cigarette usage status (*p* < 0.05).

**Table 2:** Perceived knowledge of e-cigarettes among current smokers in Al-Ahsa, KSA.

Knowledge Variables	Overall	E-Cigarette Users (n = 109)		E-Cigarette Non-Users (n = 216)			*p*-Value
	n	%	n	%	n	%	
Participants are aware of … E-cigarette	315	96.9	109	100	206	95.4	0.016
E-cigarette brands	282	86.8	106	97.2	176	81.5	<0.001
How e-cigarettes work	223	68.6	100	91.7	123	56.9	<0.001
Health consequences of e-cigarette use	196	60.3	71	65.1	125	57.9	0.126
Ingredients in e-cigarettes	175	53.8	77	70.6	98	45.4	<0.001
The nicotine level in e-cigarettes	221	68.0	96	88.1	125	57.9	<0.001
Addictive properties of e-cigarettes	118	36.3	49	45.0	69	31.9	0.015
Mean knowledge score	4.71 ± 1.79	5.52 ± 1.18		4.29 ± 1.19			<0.001

The mean perceived knowledge score of e-cigarette users was 5.52 (±1.18), with the scores ranging from 2 to 7 points. The mean perceived knowledge score of e-cigarette non-users was 4.29 (±1.19), which is lower than the cutoff point that was specified for good knowledge (5.25). Exactly 67.3% of the study participants showed a lower level of perceived e-cigarette knowledge, with scores falling below the cutoff point (5.25).

### 3.3. Factors Associated with E-Cigarette Use

Among the study participants, a third (109; 33.5%) were using e-cigarettes. When e-cigarette users were compared according to sociodemographic characteristics, a higher proportion of e-cigarette smoking was observed among the age group of 23–27 years (45; 41.3%); married (93; 85.3%); low-income group (<4000 SR/1064 USD) (61; 57.0%); and students (52; 47.7%). However, only the association between e-cigarette smoking status and occupation was found to be statistically significant (*p* = 0.007) (Table 3).

**Table 3:** Association between e-cigarette smoking status and socio-demographic variables.

Variables	E-Cigarette Users (n = 109)		E-Cigarette Non-Users (n = 216)		Total		*p*-Value
	n	%	n	%	n	%	
Age group							
18–22	45	41.3	75	34.7	120	36.9	0.228
23–27	45	41.3	85	39.4	130	40	
28–32	12	11.0	41	19.0	53	16.3	
>32	7	6.4	15	6.9	22	6.8	
Marital status							
Married	93	85.3	178	82.4	54	16.6	0.309
Single	16	14.7	38	17.6	271	83.4	
Education							
Intermediate school	1	0.9	10	4.6	11	3.4	0.309
High school	56	51.4	101	46.8	155	47.7	
Diploma	22	20.2	49	22.7	71	21.8	
Bachelor and postgraduate	30	27.5	56	25.9	88	27.1	
Income group							
<4000 SR (164 USD)	61	57.0	94	43.7	155	47.7	0.108
4000–7999 SR (164–2128 USD)	24	22.4	74	34.4	98	30.2	
8000–11,999 SR (2128–3192 USD)	15	14.0	33	15.3	48	14.8	
>11,999 SR (>3192 USD)	7	6.5	14	6.5	21	6.5	
Occupation							
Unemployed	14	12.8	15	6.9	29	8.9	0.007
Student	52	47.7	72	33.3	124	38.2	
Government employee	13	11.9	31	14.4	44	13.5	
Private employee	28	25.7	83	38.4	111	34.2	
Business	2	1.8	15	6.9	17	5.2	

### 3.4. Patterns of E-Cigarette Usage

Among the e-cigarette users, 38.5% (42/109) used e-cigarettes for more than 21 days during the past 30 days, and 34.9% (38/109) described themselves as regular users, while more than half (62.4%; 68/109) described themselves as dual users. E-cigarette users reported that the average conventional cigarette consumption reduced from 17 to 7 cigarettes per day since they started using e-cigarettes.

The most common reason stated for starting e-cigarettes was to reduce the number of cigarettes that people were smoking (71/109; 65.1%), enjoy flavors (63/109; 57.8%), cheaper than conventional tobacco cigarettes (42/109; 38.5%), and curiosity (20/109; 18.5%).

We found that the majority of the e-cigarette users (84/109; 77.1%) started e-cigarette smoking to quit conventional cigarette smoking. Among the e-cigarette users, 30.3% (33/109) felt that it was hard to quit e-cigarettes, while 41.3% (45/109) of them tried to quit using e-cigarettes in the past 30 days (Table 4). The results also show that the average number of days of electronic cigarette use among its users in the past 30 days was 16.7 (±11.3) days, the average number of hours of daily e-cigarette use was 7.7 (±5.1) h, and the average number of conventional cigarettes used with electronic cigarettes was 7.1 (±7) cigarettes/day.

**Table 4:** Use of e-cigarettes by selected characteristics of current adult cigarette smokers.

Variables	Participants	
	n	% (95% CI)
Used e-cigarette in the past 30 days (n = 325)		
No	216	66.5 (66.6–71.4)
Yes	109	33.5 (28.6–38.4)
Days of e-cigarette use in the past 30 days (n = 109)		
<7	38	34.9 (29.9–39.9)
7–14	17	15.6 (11.8–19.4)
15–21	12	11.0 (7.7–14.3)
>21	42	38.5 (33.4–43.6)
Hours of use per day (n = 109)		
<5 h	57	52.3 (47.1–57.5)
5–8 h	8	7.3 (4.6–10.0)
9–12 h	29	26.6 (22.0–31.2)
>12 h	15	13.8 (10.2–17.4)
E-cigarette use pattern (n = 109)		
Occasional	71	65.1 (60.1–70.1)
Regular	38	34.9 (29.9–39.9)
Daily users of both conventional and e-cigarettes (n = 109)		
No	41	37.6 (32.5–42.7)
Yes	68	62.4 (57.3–67.5)
Reasons for e-cigarette use (n = 109) *		
Cheaper than tobacco cigarettes	42	38.5 (33.4–43.6)
Curiosity	20	18.3 (14.3–22.3)
Reduce the number of cigarettes that I smoke	71	65.1 (60.1–70.1)
Less nicotine	17	15.6 (11.8–19.4)
Enjoying flavors	63	57.8 (52.6–63.0)
Habit	14	12.8 (9.3–16.3)
Using e-cigarettes as a strategy to quit smoking (n = 109)		
Yes	84	77.1 (72.7–81.5)
No	25	22.9 (18.5–27.3)
Tried quitting e-cigarettes in the past 30 days (n = 109)		
Yes	45	41.3 (36.2–46.4)
No	64	58.7 (53.6–63.8)
E-cigarettes are hard to quit (n = 109)		
Yes	33	30.3 (25.5–35.1)
No	76	69.7 (64.9–74.5)

* Multiple responses were allowed.

### 3.5. Predictors of E-Cigarette Use

Table 5 presents the results of uni-variable and multi-variable logistic regression analyses. We observed evidence of a statistically significant association (*p* < 0.05) between e-cigarette use and knowledge perception of e-cigarettes, occupation, and conventional cigarette smoking pattern of the current smokers. Our results suggest that current smokers with a good perceived knowledge of e-cigarettes were more likely to use e-cigarettes than those with a poor level of knowledge (OR: 3.49; 95% CI: 2.07–5.90). Compared to unemployed current smokers, private employees (OR: 0.25; 95% CI: 0.07–0.85) and individuals engaged in free business (OR: 0.09; 95% CI: 0.01–0.63) were less likely to use e-cigarettes. We also found that e-cigarette use was strongly associated with cigarette smoking patterns (*p* < 0.001). The proportion of e-cigarette users was much higher among occasional smokers than regular smokers (OR: 2.28; 95% CI: 1.17–4.41). However, we did not observe any evidence of a statistically significant association between e-cigarette use and marital status, age group, level of education, income group, and the number of conventional cigarettes smoked per day. 

**Table 5:** Factors associated with e-cigarette use among current smokers in Al–Ahsa, KSA.

Factors		Odds Ratio (95% CI)	
		Uni-Variable	Multi-Variable
Knowledge	Poor	1	
	Good	3.34 (2.07–5.39) *	3.49 (2.07–5.90) *
Marital status	Married	1	
	Single	0.81 (0.43–1.52)	1.47 (0.60–3.59)
Educational level	Intermediate school	1	
	High school	5.35 (0.67–42.88)	4.29 (0.47–39.18)
	Diploma	4.49 (0.54–37.27)	4.80 (0.52–44.79)
	Bachelor and postgraduate	5.71 (0.69–46.71)	5.58 (0.60–52.29)
Occupation	Unemployed	1	
	University student	0.77 (0.34–1.74)	0.87 (0.32–2.34)
	Government employee	0.45 (0.17–1.19)	0.27 (0.06–1.16)
	Private employee	0.36 (0.16–0.84) *	0.25 (0.07–0.85) *
	Free business	0.14 (0.03–0.74) *	0.09 (0.01–0.63) *
Monthly income	<4000 SAR	1	
	4000–7999 SAR	0.50 (0.29–0.88) *	1.11 (0.42–2.90)
	8000–11,999 SAR	0.70 (0.35–1.40)	1.69 (0.50–5.69)
	>11,999 SAR	0.77 (0.24–2.02)	2.19 (0.50–9.59)
Age group	18–22 years	1	
	23–27 years	1.29 (0.49–3.39)	1.09 (0.54–2.19)
	28–32 years	1.13 ( 0.43–2.98)	0.83 (0.29–2.35)
	>32 years	0.63 (0.21–1.89)	1.21 (0.29–5.05)
Conventional cigarette smoking pattern	Regular smoker	1	
	Occasional smoker	2.45 (1.47–4.07) *	2.28 (1.17–4.41) *
Conventional cigarette smoked/day	<9 cigarettes	1	
	9–16 cigarettes	0.74 (0.37–1.46)	1.12 (0.49–2.57)
	17–24 cigarettes	0.66 (0.33–1.30)	1.54 (0.63–3.80)
	>24 cigarettes	0.46 (0.19–1.09)	1.20 (0.41–3.51)

* *p*-value less than 0.05.

### 3.6. Predictors of Good Perceived Knowledge of E-Cigarettes

A multiple logistic regression model adjusting for age, marital status, education, income and occupation shows (Table 6) that participants who were aware of the health consequences of e-cigarette use were 13.3 times more likely to have a good level of perceived knowledge of e-cigarettes (OR: 13.3; 95% CI: 6.96–25.41) when compared with individuals who were not aware of the health consequences of e-cigarette use. The other covariates that showed a positive significant association with good perceived knowledge of e-cigarettes were the use of e-cigarettes (OR: 3.6, 95% CI: 2.14–5.98), knowing that e-cigarettes have addictive properties (OR: 5.1; 95% CI: 3.1–8.5), daily users of conventional and e-cigarettes (OR: 2.86; 95% CI: 1.61–5.10), trying to quit e-cigarettes in the past 30 days (OR: 2.17; 95% CI: 1.1–4.25), and believing that quitting e-cigarettes is hard (OR: 2.02; 95% CI: 1.17–3.49).

**Table 6:** Factors associated with good perceived knowledge of e-cigarettes among current smokers in Al–Ahsa, KSA.

Variables (Reference Category)	n (%)	Odds Ratio (95% Confidence Interval)	
		Uni-Variable Analysis	Multi-Variable Analysis ^
Use e-cigarette (no)	109 (33.5)	3.34 (2.07–5.40) **	3.57 (2.14–5.98) **
Use e-cigarette to quit smoking (no)	75 (68.8)	1.11 (0.49–2.54)	1.31 (0.52–3.34)
Aware of the health consequences of e-cigarette use (no)	196 (60.3)	11.75 (6.38–21.61) **	13.30 (6.96–25.41) **
Aware that e-cigarette has addictive properties (no)	118 (36.3)	5.26 (3.22–8.58) **	5.14 (3.10–8.52) **
Daily users of both e-cigarettes and conventional cigarettes (no)	68 (62.4)	2.90 (1.67–5.03) **	2.86 (1.61–5.10) **
Tried to quit e-cigarettes in the past 30 days (no)	45 (41.3)	2.20 (1.16–4.17) *	2.17 (1.1–4.25) *
Believe that quitting e-cigarettes is hard (no)	33 (30.3)	2.1 (1.23–3.55) *	2.02 (1.17–3.49) *

* *p* < 0.05; ** *p* < 0.001; ^ adjusted for age, marital status, income and occupation.

## 4. Discussion

This study aimed to assess the use of e-cigarettes among male current smokers in Al-Ahsa KSA. The findings of the study suggest that there is a high prevalence of e-cigarette use among Saudi male smokers. Occasional smokers, knowledge perception of e-cigarettes, and occupation type were found to have a significant association with the use of e-cigarettes among current smokers.

Regarding the prevalence of e-cigarette use among current smokers, we found that one-third of the participants (109; 33.5%) used e-cigarettes in the past 30 days. Similar to our study findings, a survey among university student smokers in Riyadh found a much higher prevalence (54.4%) of e-cigarette use [35]. A high prevalence of e-cigarette use has also been reported in the USA [32]. This popularity of e-cigarette use is probably due to widespread e-cigarette promotion, increasing awareness about the health hazards of conventional cigarettes, and local e-cigarettes or tobacco-regulation policies [16,32]. For example, to date, in the KSA, there are no restrictions on advertising, promotion, and sponsorship of e-cigarettes, or decision made to regulate e-cigarettes. The use of e-cigarettes is prohibited where smoking is prohibited, health warnings are required, and the law prohibits specific elements, such as certain flavors, vitamins, caffeine, additives having coloring properties, and additives that contribute to toxicity. In Hong Kong, e-cigarettes have been forbidden [36] and are considered tobacco products in the USA [37]. These findings demand urgent policy-level actions to curb the growing rates of e-cigarette use among the current male smokers in the KSA. 

In this study, the perceived knowledge of e-cigarettes among current male smokers was high. Similar higher rates have been observed in earlier studies conducted in Italy [38] and Hong Kong [39]. A meta-analysis of 28 studies showed that the global average e-cigarette awareness is 61.2%, which is much lower compared to our study [40]. In summary, our findings indicate that the level of awareness of the participants is higher than the regional and global average level of awareness about e-cigarettes. The different findings are explained by the difference in economic development, culture and population characteristics, and widespread use of social media. Our findings indicated that the majority of e-cigarette users have a good perceived knowledge of e-cigarettes, which is consistent with earlier studies on knowledge about e-cigarettes among young adults [32,41]. This also explains the high level of e-cigarette awareness among current adult smokers in the KSA.

Our study found that the intention to reduce or quit conventional cigarette smoking, cost, recreation, and curiosity are the key reasons for using e-cigarettes. These findings are comparable to previous study findings from the USA [42] and China [31]. The Centers for Disease Control and Prevention National survey—2016 revealed that the main reasons behind using e-cigarettes are friends and family members using them, available in flavors, and less harmful than other forms of tobacco cigarettes [43]. Our findings support a distinction between intention and non-intentional-oriented reasons for use, while the last two reasons are less likely to lead to continued use of e-cigarettes.

Regarding the relationship between e-cigarette use and consumption of conventional cigarettes, the results indicated that the rate of conventional cigarettes smoked per day was significantly lower among male e-cigarette users compared to non-e-cigarettes users. This finding suggests that e-cigarette use reduces conventional smoking; however, there could be alternative explanations, including the initiation of a small number of cigarettes following the use of e-cigarettes. A randomized control trial and a cross-sectional survey conducted in Italy found that e-cigarette use reduces conventional smoking [12,44]. However, we could not establish a causal relationship between e-cigarette use and reductions in conventional cigarette smoking because of our study’s inherent design limitations.

In our study, most e-cigarette users reported that e-cigarette use helped them reduce the number of conventional cigarettes they smoke per day. This finding is consistent with a study conducted in France, which reported that e-cigarette use is associated with a significant reduction in the number of cigarettes smoked per day [31,45]. This is also congruent with the evidence from a systematic review and meta-analysis conducted by Rahman et al. [46], which reported that the use of e-cigarettes is associated with a reduction in the number of cigarettes used. 

Our study findings indicate that e-cigarette use could be a helpful tool to reduce the use of conventional smoking. This result is supported by Qanash et al. [6] who reported that e-cigarettes help smokers to quit smoking. In contrast, Olfson et al. [47] stated that e-cigarette use does not help to quit tobacco smoking among young adults in the USA. Further, results of a recent systematic review and meta-analysis showed that e-cigarette use is not associated with smoking cessation [18,19]. These contradictory findings indicate that further studies are needed to determine the effectiveness of e-cigarette use in quitting smoking.

This study had several limitations. Although we tried to recruit representative samples, including different demographics and socioeconomic status, from three administrative areas in Al-Ahsa province, our study participants were skewed towards younger educated individuals; though these two variables (education level and age) did not illustrate any significant association with the knowledge score, nor the e-cigarette use, this might not affect the generalizability of our results. Additionally, the higher perceived knowledge of e-cigarettes among the study participants might not be an accurate reflection of their knowledge of e-cigarettes and could be because of the excessive use of e-cigarettes. There are other limitations, for example, statistical limitations in establishing causality, and controlling for confounders, such as nicotine dependence. This study was conducted on male current smokers only, which limits the generalizability of the study findings only to male smokers. Despite these limitations, this study addresses an important knowledge gap in the research area by assessing the prevalence of e-cigarette use among adult male current smokers and determining the associations between e-cigarette use and socio-demographic variables. This study provides useful data on the effectiveness of e-cigarettes in reducing conventional cigarettes. However, readers should note that our finding on cessation of smoking because of e-cigarette use is primarily based on the participants’ reports of the number of cigarettes they cut down by using e-cigarettes.

## 5. Conclusions

The use and perceived knowledge of e-cigarettes are higher among current male smokers in the Al-Ahsa province, KSA. Current smokers reported using e-cigarettes for a variety of reasons, such as curiosity, enjoying flavors, being cheaper than conventional cigarettes, and intention to reduce or quit conventional cigarette smoking. We observed an association between e-cigarette use and good knowledge perception of e-cigarettes, conventional cigarette-smoking patterns, and occupation of the current smokers of conventional cigarettes. Our study findings suggest that e-cigarette use could be a helpful tool to reduce the use of conventional smoking. However, further studies are needed to determine the population-level impact of e-cigarette usage on smoking cessation. In addition, preventive efforts focusing on potentially harmful and addictive properties of e-cigarettes are essential. 

## Data Availability

The datasets used during the current study are available from the PI (YEM) and are available on reasonable request.

## Abbreviations

E-cigarette	Electronic cigarette
KSA	Kingdom of Saudi Arabia
SAR	Saudi Arabian Riyal
OR	Odds Ratio
CI	Confidence Interval
